# Chinese herbal medicine for migraine management: A hospital-based retrospective analysis of electronic medical records

**DOI:** 10.3389/fmed.2022.936234

**Published:** 2022-11-10

**Authors:** Shaohua Lyu, Claire Shuiqing Zhang, Jingbo Sun, Heng Weng, Charlie Changli Xue, Xinfeng Guo, Anthony Lin Zhang

**Affiliations:** ^1^The China Australia International Research Centre for Chinese Medicine, School of Health and Biomedical Sciences, STEM College, RMIT University, Bundoora, VIC, Australia; ^2^Guangdong Provincial Hospital of Chinese Medicine and Guangdong Provincial Academy of Chinese Medical Sciences, The Second Affiliated Hospital of Guangzhou University of Chinese Medicine, Guangzhou, China

**Keywords:** migraine, electronic medical records (EMR), Chinese medicine, Chinese herbal medicine (CHM), real-world, clinical features, treatment patterns, therapeutic characteristics

## Abstract

**Background:**

Migraine is a chronic neurological disease causing significant socioeconomic burden and impaired quality of life. Chinese medicine is commonly used for migraine in China. Clinical trials have generated evidence of the effectiveness of Chinese medicine therapies for migraine. However, little is known about how to use these therapies to treat migraine in real-world clinical settings.

**Methods:**

In this retrospective study, we analyzed data from the electronic medical records (EMRs) of 2,023 migraine patients who attended the Guangdong Provincial Hospital of Chinese Medicine (GPHCM) between July 2018 and July 2020.

**Results:**

More than three-quarters (77.21%) of the patients were female. Most (78.20%) of the patients were aged between 18 and 50 years, 18.49% were aged above 50 years, and the remaining 3.31% were under 18 years. Sleep disorders were the most documented comorbidity occurring in 27.29% of patients, and more common in females (29.77%) than male (18.87%). Fatigue was the most frequently reported trigger of migraine attacks among all patients (9.39%), while menstruation was the most common trigger for female patients (10.24%). Less than a quarter of patients (21.01%) reported a history of taking analgesic medication for their migraine. The median treatment duration reported by the patients was 10 days. Chinese herbal medicine (CHM) was the predominant treatment for migraine at the hospital (88.48%), while pharmacotherapies were prescribed to 28.97% of the patients. CHM was prescribed more often as a sole treatment (53.58% of patients) than combined with pharmacotherapies (27.39% of patients). Among patients who reported improvements after taking CHM, the most frequently used herbs were *fu ling* and *chuan xiong*, the most frequent patented CHM product was *tong tian* oral solution, and the main herbal formulae were *chuan xiong cha tiao san* and *yi qi cong ming tang*.

**Conclusion:**

CHM formulae, such as *chuan xiong cha tiao san* and *yi qi cong ming tang*, patented CHM product *tong tian* oral solution, and some herbs are potentially effective treatments for migraine. As such, CHM can be used as an alternative to conventional pharmacotherapies for migraine and is worth further evaluation in randomized controlled trials.

## Introduction

Migraine is a prevalent primary headache disorder characterized by recurrent, unilateral, moderate-to-severe pulsating headaches (1). The headache is usually associated with nausea, vomiting, phonophobia, and photophobia (1). Migraine is often accompanied by comorbidities, such as sleep disorders, anxiety, and depression (2–6), and can be triggered by common lifestyle factors, including stress, caffeine, and menstruation (7, 8). According to a systematic review of the Global Burden of Disease Study (9), migraine was estimated to affect 1.04 billion people with a global age-standardized prevalence of 14.4% and caused 45.1 million years of life with disabilities (YLDs). Both the prevalence and YLDs of migraine peaked between 35 and 39 years of age in both genders (9).

Current pharmacotherapies for migraine consist of acute treatments including triptans and ergots, and prophylactic therapies such as calcium channel blockers (CCBs), beta-blockers, and calcitonin gene-related peptide (CGRP) antibodies (10, 11). Patients are often unsatisfied with these medications due to their insufficient treatment effects, potential risk of causing comorbidities, and unwanted side effects (12–14). Moreover, acute pain-relief medications tend to be overused by migraine patients, resulting in the transformation from episodic migraine to chronic migraine (15, 16) as well as a higher risk of psychological comorbidities (17, 18), and hence increases the disease burden (19).

Due to these challenges, complementary and alternative medicine is popular among migraine patients (20, 21). Chinese medicine therapies, including Chinese herbal medicine (CHM) and acupuncture, were reported to be prescribed over 60% of migraine patients in China (22). Systematic reviews concluded that Chinese medicine therapies were comparable or superior to conventional pharmacotherapies, either being used solely or in combination with pharmacotherapies (23–33). However, the existing research evidence was obtained from randomized controlled trials (RCTs) that applied unified treatments to certain populations based on pre-defined selection criteria. There is a lack of real-world information about treatment patterns and first-hand clinicians’ experience. Moreover, the clinical characteristics and preferences of migraine patients seeking Chinese medicine therapies also remain unclear. Therefore, we conducted a retrospective analysis of electronic medical records (EMRs) from a large-sized Chinese medicine hospital to explore and summarize the real-world clinical evidence, patients’ characteristics, and clinicians’ experiences. The results of this research will be valuable for evidence-based clinical practice.

## Materials and methods

This retrospective study collected clinical data from outpatient departments at the Guangdong Provincial Hospital of Chinese Medicine (GPHCM), the largest tertiary hospital that provides integrated Chinese medicine and conventional therapies for patients in China (34).

### Ethics consideration

The study was approved by the Human Research Ethics Committee (HREC) of GPHCM (ZE2020-243-01) and registered with the HREC at RMIT University (no. 24235). Informed consent was waived since identifiable information, including names, identification numbers, dates of birth, phone numbers, and residential addresses of patients, had been deidentified in the dataset before data screening and analyses.

### Data search and screening

The EMRs between July 2018 and July 2020 were retrieved by the Information Technology Department of GPHCM to locate migraine-related patient encounters (PEs). Only those PEs with migraine as the primary diagnosis were exported to an Excel dataset and then screened by a clinician who specialized in headache and neurology (S Lyu) for eligibility.

PE records were excluded if they met any of the following criteria: (1) incomplete records; (2) symptoms not consistent with migraine diagnostic criteria (1); (3) chief complaint is not related to migraine; and (4) reporting treatment effects in the first PE among a series of PEs. Uncertainties were resolved by consulting a senior headache/neurology specialist (J Sun).

### Data extraction

Preliminary data extraction was conducted by H Weng using TNorm, a rule-based and pattern learning-based approach developed for automatic temporal expression extraction and normalization for data in Chinese text (35). Structured data, such as age (at the first visit), date of visits, gender, current and previous medical histories, diagnoses, and treatment details, were extracted from each PE into an Excel dataset at this stage. During this procedure, PEs sharing the same medical record number were merged as one EMR.

Further data extraction was manually conducted by S Lyu to identify unstructured data on migraine comorbidities, triggers, numbers of visits, total treatment duration, patients’ response to treatments, and in which visits patients reported improvements. Clinical conditions, such as depression, anxiety, sleep disorder, rhinitis, and dermatological conditions, were considered as comorbidities of migraine according to previous research (36–40). Factors including fatigue, menstruation, coldness or wind, emotion, crowded environment, poor sleep, weather changes, stress, diet, strong light exposure, exercise, washing hair, and odor, were classified as triggers of migraine episodes based on clinical guidelines and previous studies (41–48). Since migraine triggers vary across individuals, the trigger data were extracted based on patient-reported information. Treatment responses were briefly classified as “improved” and “no response or unclear,” based on patients’ self-reporting as recorded in EMRs. Where EMRs recorded a response consistent with the Diagnostic Criteria and Category of Treatment Response of *Toufeng* in China (49), including the reduction of migraine or other symptoms in general after certain treatments, the patients were marked as “improved” and the previous PE was marked as “patient encounter reporting improvements (PERI).” Data on the CHM prescribed in PERIs, including Chinese herbal decoctions and patented Chinese herbal medicine products (PCHMP), were extracted for further analyses. Acupuncture points were not extracted since they usually were not recorded in detail.

Logical data checking and correction were also conducted by S Lyu to identify inconsistencies between PEs from the same EMR.

### Data standardization

Descriptions of the migraine-associated symptoms, comorbidities, triggers, and treatment responses in the EMRs were standardized using common medical terminologies. Chinese medicinal herbs in granule or decoction forms, which were processed in various ways were standardized as one herb using the herb name listed in the China Pharmacopoeia (*version 2020*) (50). For example, *chao bai zhu* (fried *bai zhu*) was standardized as *bai zhu* since they were not distinguished from each other in the China Pharmacopoeia. In contrast, *sheng di huang* remains distinguished from *shu di huang* because both names are listed in the China Pharmacopoeia with different therapeutic functions (50); this rule was also applied for *gan cao* and *zhi gan cao*, and *ban xia* and *sheng ban xia*.

### Data analyses

Categorical variables were presented as frequency/percentage and compared *via Chi-*square or Fisher’s tests, where appropriate. Continuous variables were presented as mean with standard deviation and compared *via* the *t*-test when they were normally distributed. Otherwise, they were presented as median with interquartile range and compared *via* the Mann–Whitney *U*-test. SPSS software (*version 20.0, SPSS Inc., Chicago, IL, U.S.A*) was used for the descriptive analyses of patients’ characteristics and treatment information. Values of *p* < 0.05 were considered to indicate statistical significance.

Association rule construction based on the Apriori algorithm (51, 52) was conducted to identify high-frequency herb combinations and associations between triggers or comorbidities with herbs, using SPSS Modeler software (*version 18.0, SPSS Inc., Chicago, IL, U.S.A*). A network diagram was generated to visualize the co-occurrence between the frequently used herbs.

Generally, an Apriori algorithm is constructed based on the notion that the antecedent item sets and the consequent item set only co-occur in the dataset rather than due to a causal effect (53). Three parameters—support, confidence, and lift—are used to assess the associations of variables. Support is the prevalence of antecedent and corresponds to statistical significance (see formula A) (51). The minimum threshold of support is usually predefined to avoid occasional co-occurrence (51, 52). After iterative tests, we set the support level at 5% for herb pair and herb combination analyses to ensure only frequently used herbs appear in the antecedent. Confidence and lift indicate the strength of association, with higher values showing more robust connections between the consequent and antecedent. Confidence reflects the possibility of co-occurrences of consequent and antecedent in the datasets consisting of antecedent (see formula B), while the lift is a value that represents the likelihood of an increase in the consequent given a particular antecedent (see formula C). The thresholds of confidence in the association rules were determined according to individual situations.

The formulae for these three metrics are listed as follows (53, 54):


(A) $$\mathrm{Support}:s\left(X\to Y\right)=P\left(X\cup Y\right)=\frac{\sigma \left(X\cup Y\right)}{N}$$

(B) $$\mathrm{Confidence}:c\left(X\to Y\right)=\frac{P\left(X\cup Y\right)}{P\left(X\right)}=\frac{\sigma \left(X\cup Y\right)}{\sigma \left(X\right)}$$

(C) $$\mathrm{Lift}:l\left(X\to Y\right)=\frac{P\left(X\cup Y\right)}{P\left(X\right)*P\left(Y\right)}=\frac{\sigma \left(X\cup Y\right)}{\sigma \left(X\right)*\sigma \left(Y\right)}*N$$

## Results

### Summary of the research procedure

**FIGURE 1:**
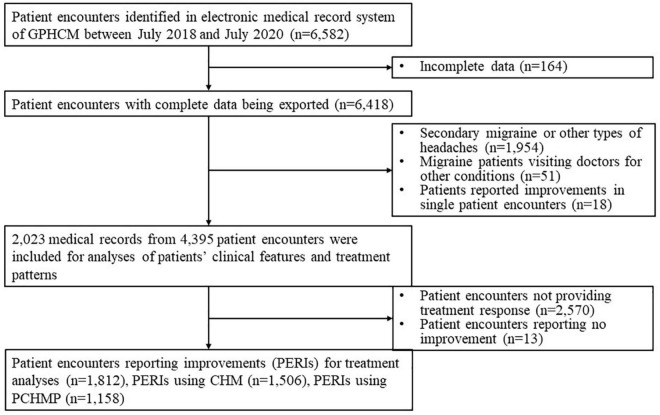
Flow chart of the study procedure. CHM, Chinese herbal medicine; GPHCM, Guangdong Provincial Hospital of Chinese Medicine; PERI, Patient encounter reporting improvements; PCHMP, Patented Chinese herbal medicine products.

A total of 6,582 PEs with a primary diagnosis of migraine were identified and exported. After eligibility screening, 4,395 PEs from 2,023 EMRs (i.e., individual patient records) were included and analyzed for patient characteristics and treatment patterns. Among them, 1,812 PERIs contributed to the in-depth analyses of treatment patterns and therapeutic characteristics to identify the frequently used herbs and herb combinations. The procedure is illustrated in Figure 1.

### Clinical features of all patients

#### Demographics

Among the 2,023 patients (EMRs), 1,562 (77.21%) were female. The mean age of all patients was (37.89 ± 12.74) years. The mean age of female patients (38.11 ± 12.53 years) was similar to that of male patients (37.16 ± 13.42 years) (*p* = 0.16). More than three-quarters of the patients were adults under 50 years of age (*n* = 1,582, 78.20%), while 374 (18.49%) patients were over 50 years of age and the remaining 67 (3.31%) patients were under 18 years of age (Table 1).

**TABLE 1:** Characteristics, comorbidities and triggers of the patients.

			Overall (*n* = 2, 023)	Male (*n* = 461)	Female (*n* = 1, 562)
			
		Mean age, years (SD)	37.89 (12.74)	37.16 (13.42)	38.11 (12.53)
		Categories	No (%)		
Characteristics	Age group	<18	67(3.31)	26 (5.64)	41 (2.62)
		18 ≤ age < 50	1,582 (78.20)	357 (77.44)	1,225 (78.43)
		≥50	374 (18.49)	78 (16.92)	296 (18.95)
	Aura	Migraine with aura	100 (4.94)	25 (5.42)	75 (4.80)
		Migraine without aura	40 (1.98)	5 (1.08)	35 (2.24)
		NS	1,883 (93.08)	431 (93.49)	1,452 (92.96)
	Family history	Yes	225 (11.12)	45 (9.76)	180 (11.52)
		No	84 (4.15)	23 (4.99)	61 (3.91)
		NS	1,714 (84.73)	393 (85.25)	1,321 (84.57)
	Acute medication usage	Yes	425 (21.01)	108 (23.43)	317 (20.29)
		No	7 (0.34)	3 (0.65)	4 (0.26)
		NS	1,591 (78.65)	350 (75.92)	1,241 (79.45)
	Childbearing* (valid *n* = 1,505)	Yes	65 (4.32)	N/A	65 (4.32)
		No	0 (0)	N/A	0 (0)
		NS	1,440 (95.68)	N/A	1,440 (95.68)
Comorbidities	Anxiety and/or depression	Yes	117 (5.78)	20 (4.34)	97 (6.21)
		No	0 (0)	0 (0)	0 (0)
		NS	1,906 (94.22)	441 (95.66)	1,465 (93.79)
	Rhinitis	Yes	15 (0.74)	5 (1.08)	10 (0.64)
		No	0 (0)	0 (0)	0 (0)
		NS	2,008 (99.26)	456 (98.92)	1,552 (99.36)
	Dermatological conditions	Yes	37 (1.83)	5 (1.08)	32 (2.05)
		No	0 (0)	0 0)	0 (0)
		NS	1,986 (98.17)	456 (98.92)	1,530 (97.95)
	Sleep disorders	Yes	552 (27.29)	87 (18.87)	465 (29.77)
		No	338 (16.71)	80 (17.35)	258 (16.52)
		NS	1,133 (56.00)	294 (63.77)	839 (53.71)
Triggers	Fatigue	Yes	190 (9.39)	47 (10.20)	143 (9.15)
		No	24 (1.19)	3 (0.65)	21 (1.34)
		NS	1,809 (89.42)	411 (89.15)	1,398 (89.50)
	Coldness or Wind	Yes	99 (4.89)	25 (5.42)	74 (4.74)
		No	26 (1.29)	4 (0.87)	22 (1.41)
		NS	1,898 (93.82)	432 (93.71)	1,466 (93.85)
	Emotion	Yes	77 (3.81)	14 (3.04)	63 (4.03)
		No	24 (1.19	2 (0.43)	22 (1.41)
		NS	1,922 (95.00)	445 (96.53)	1,477 (94.56)
	Crowded environment	Yes	76 (3.76)	20 (4.34)	56 (3.59)
		No	27 (1.33)	4 (0.87)	23 (1.47)
		NS	1,920 (94.91)	437 (94.79)	1,483 (94.94)
	Poor sleep	Yes	68 (3.36)	9 (1.95)	59 (3.78)
		No	27 (1.33)	4 (0.87)	23 (1.47)
		NS	1,928 (95.30)	448 (97.18)	1,480 (94.75)
	Weather changes	Yes	48 (2.37)	14 (3.04)	34 (2.18)
		No	27 (1.33)	4 (0.87)	23 (1.47)
		NS	1,948 (96.29)	443 (96.10)	1,505 (96.35)
Characteristics	Stress	Yes	26 (1.29)	11 (2.39)	15 (0.96)
		No	27 (1.33)	4 (0.87)	23 (1.47)
		NS	1,970 (97.38)	446 (96.75)	1,524 (97.57)
	Diet	Yes	21 (1.04)	12 (2.60)	9 (0.58)
		No	26 (1.29)	3 (0.65)	23 (1.47)
		NS	1,976 (97.68)	446 (96.75)	1,530 (97.95)
	Strong light exposure	Yes	18 (0.89)	4 (0.87)	14 (0.90)
		No	27 (1.33)	4 (0.87)	23 (1.47)
		NS	1,978 (97.78)	453 (98.26)	1,525 (97.63)
	Exercise	Yes	11 (0.54)	3 (0.65)	8 (0.51)
		No	27 (1.33)	4 (0.87)	23 (1.47)
		NS	1,985 (98.12)	454 (98.48)	1,531 (98.02)
	Washing hair	Yes	8 (0.40)	0 (0)	8 (0.51)
		No	26 (1.29)	4 (0.87)	22 (1.41)
		NS	1,989 (98.32)	457 (99.13)	1,532 (98.08)
	Odor	Yes	1 (0.05)	1 (0.22)	0 (0)
		No	27 (1.33)	4 (0.87)	23 (1.47)
		NS	1,995 (98.62)	456 (98.92)	1,539 (98.53)
	Menstruation^#^ (valid *n* = 1,562)	Yes	160 (10.24)	N/A	160 (10.24)
		No	52 (3.33)	N/A	52 (3.33)
		NS	1,350 (86.43)	N/A	1,350 (86.43)

*Calculated in female adults (age > 18).

^#^Calculated in female patients.

N/A, not applicable; No., number; NS, not stated; SD, standard deviation.

#### Characteristics

The characteristics of migraine were insufficiently recorded in the EMRs. Only 140 (6.92%) EMRs contained information about aura, with 100 (4.94%) patients diagnosed as migraine with aura and 40 (1.98%) were diagnosed as migraine without aura. A total of 225 (11.12%) patients had a family history of migraine, 84 (4.15%) patients did not have such a family history, and the remaining 1,714 EMRs did not include this information. Acute medication-taking behavior was only reported by 432 (21.35%) patients. Among these patients, 425 (21.01%) had taken acute medication for migraine and 7 patients reported never taking any acute medication. The remaining 1,591 EMRs did not provide clear information on the history of acute medication usage history (Table 1).

In addition, 65 (4.32%) adult female patients reported the occurrence or aggregation of migraine after childbearing. However, the number of adult women whose migraine were not associated with childbearing was unavailable (Table 1).

#### Comorbidities

Common migraine comorbidities that patients reported were sleep disorders (*n* = 552, 27.29%), anxiety and/or depression (*n* = 117, 5.78%), rhinitis (*n* = 15, 0.74%), and dermatological conditions (*n* = 37, 1.83%).

As the most reported comorbidity, sleep disorders involved symptoms of insomnia, interrupted sleep, dreaminess, and so on. Around half (*n* = 890, 43.99%) of the patients were recorded with information on their sleep quality, with 338 (16.71%) reporting satisfactory sleep quality. Based on the available data, female patients seemed more likely to have sleep disorders than male patients (29.77% vs. 18.87%) (Table 1).

#### Triggers

Migraine triggers documented in the PEs for both genders were fatigue (*n* = 190, 9.39%), coldness (*n* = 99, 4.89%), emotion (*n* = 77, 3.81%), crowded environment (*n* = 76, 3.76%), poor sleep (*n* = 68, 3.36%), weather changes (*n* = 48, 2.37%), stress (*n* = 26, 1.29%), diet (*n* = 21, 1.04%), strong light exposure (*n* = 18, 0.89%), exercise (*n* = 11, 0.54%), washing hair (*n* = 8, 0.40%), and odor (*n* = 1, 0.05%). Among female patients, menstruation was the leading trigger (*n* = 160, 10.24% of all female patients) (Table 1).

### Treatment patterns of all patients

#### Treatment categories

The treatment patterns among 2,023 EMRs are presented in Table 2. Most migraine patients (*n* = 1,790, 88.48%) had been prescribed CHM, followed by conventional pharmacotherapies (*n* = 586, 28.97%) and acupuncture (*n* = 348, 17.20%). CHM was prescribed more often as a sole treatment (*n* = 1,084) than in combination with conventional pharmacotherapies (*n* = 554). There was no significant statistical difference in treatment methods between genders (*p* = 0.467) nor between adult and non-adult groups (*p* = 0.668). During the treatment course, only 32 (1.58%) patients were newly prescribed acute medication, and 428 (21.16%) patients were prescribed CCBs (flunarizine or nimodipine tablets). Other pharmacotherapies included hypnotic drugs (*n* = 81, 4.00%), antidepressants/antianxiety drugs (*n* = 61, 3.02%), and anti-epileptic drugs (*n* = 18, 0.89%).

**TABLE 2:** Treatment categories.

Treatment categories		No. (%*)	Gender			Age		
			Female	Male	Difference	<18	≥18	Difference^#^
Single use	ACU related	146 (7.22)	106	40	χ^2^ = 6.647 *P* = 0.467 df = 7	3	143	χ^2^ = 4.37 *P* = 0.668 df = 7
	CHM	1,084 (53.58)	845	239		41	1,043	
	PCHMP	26 (1.29)	16	10		0	26	
Combinations	ACU related + CHM	152 (7.51)	120	32		2	150	
	ACU related +CHM+ PCHMP	44 (2.17)	34	10		1	43	
	ACU related + PCHMP	6 (0.30)	4	2		0	6	
	CHM+ PCHMP	510 (25.21)	396	114		17	493	
No treatment		55 (2.72)	41	14		3	52	
Total		2,023 (100)	1,562	461	N/A	67	1,956	N/A
CP	Acute medications	32 (1.58)	N/A					
	AEDs	18 (0.89)						
	Antidepressants/Antianxiety	61 (3.02)						
	CCB	428 (21.16)						
	Hypnotic	81 (4.00)						
	Sub-total WM	586 (28.97)						

ACU, acupuncture; AEDs, Anti-epileptic drugs; CCB, calcium channel blocker; CHM, Chinese herbal medicine; CP, conventional pharmacotherapies; PCHMP, patent Chinese herbal medicine product; N/A, not applicable; No., number.

*Percentage in all patients.

^#^Tested by Fisher’s exact test.

#### Times of visits and treatment duration

Fifty-five patients only visited the doctors for advice or examinations and did not receive any treatment. The total number of visits to the hospital for migraine was available from 1,976 patients. More than half (*n* = 1,069, 54.10%) only visited the hospital once for migraine, therefore, their responses to treatments were not available (Table 3).

**TABLE 3:** Description of treatment duration and visit times.

		Treatment duration (days)	Treatment duration to show effects (days)	Total times of visits
No. of medical records	Valid	1,968	397	1,976
	Missing	55	1,626	47
Median		10	14	1
Percentiles	25	7	7	1
	75	29	28	2
Mode		7	7	1
Frequency of mode (%*)		635 (32.3)	121 (30.48)	1,069 (54.10)

*Valid percentage; CHM, Chinese herbal medicine; No., number.

According to 1,968 EMRs with treatment information, the median duration of migraine-specific treatments was 10 days. Of these patients, 72.41% (*n* = 1,425) of patients underwent treatment for less than 4 weeks, and nearly one-third of them (*n* = 635, 31.39%) only accepted treatments for 7 days. This is notably shorter than the recommendation of “at least 4 weeks treatment for migraine prophylaxis” in clinical guidelines (10, 11) (Table 3).

In addition, the duration of the treatment which achieved certain improvements was available from 397 EMRs. The most commonly reported duration was 7 days (*n* = 121, 30.48%), and the median treatment duration was 14 days (Table 3).

### Therapeutic characteristics of Chinese herbal medicine

The therapeutic characteristics of CHM, as the predominant treatment method, were further summarized based on the 1,812 PERIs as follows, regardless of whether they were used alone or in combination with other treatments.

#### Chinese herbal decoction

##### Frequently used herb

In 1,506 PERIs, 258 individual herbs were prescribed 23,329 times in a decoction form. Herbs recorded by more than 15% of PERIs are listed in Table 4. The top 10 frequently used herbs were *fu ling, chuan xiong, bai shao, ban xia, zhi gan cao, gui zhi, yan hu suo, bai zhu, chen pi*, and *chang pu*.

**TABLE 4:** Frequently used herbs in Chinese herbal decoctions for which patients reported improvements.

Herb name in *pin yin* and Chinese characters	Scientific name	No. (%)
*Fu ling* 茯苓	Poria cocos (Schw.) Wolf	955 (52.70)
*Chuan xiong* 川芎	Ligusticum chuanxiong Hort.	929 (51.27)
*Bai shao* 白芍	Paeonia lactiflora Pall.	894 (49.34)
*Ban xia* 半夏	Pinellia ternate (Thunb.) Breit.	837 (46.19)
*Gan cao* (honey fried) 炙甘草	Glycyrrhizae Radix Et Rhizoma Praeparata Cum Melle	790 (43.60)
*Gui zhi* 桂枝	Cinnamomum cassia Presl	788 (43.49)
*Yan hu suo* 延胡索	Corydalis yanhusuo W.T. Wang	763 (42.11)
*Bai zhu* 白术	Atractylodes macrocephala Koidz.	761 (42.00)
*Chen pi* 陈皮	Citrus reticulata Blanco	736 (40.62)
*Chang pu* 石菖蒲/九节菖蒲	Acorus tatarinowii Schott	637 (35.15)
*Wu yao* 乌药	Lindera aggregate (Sims) Kos-term.	625 (34.49)
*Xiang fu* 香附	*Cyperus rotundus* L.	612 (33.77)
*Bai zhi* 白芷	1. Angelica dahurica (Fisch,ex Hoffm.) Benth.et Hook.f. 2. Angelica dahurica (Fisch. ex Hoffm.) Benth.et Hook.f.var.formosana (Boiss.) Shan et Yuan	572 (31.57)
*Liu ji nu* 刘寄奴	Herba Artemisiae Anomalae	546 (30.13)
*Xiao hui xiang* 小茴香	Foeniculum vulgare Mill.	536 (29.58)
*Hou po* 厚朴	1. Magnolia officinalis Rehd.et Wils. 2. Magnolia officinalis Rehd.et Wils.var.biloba Rehd.et Wils.	526 (29.03)
*Sha ren* 砂仁	1. Amomum villosum Lour. 2. Amomum villosum Lour.var.xanthioides T.L.Wu et Senjen 3. Amomum longiligulare T.L.Wu	526 (29.03)
*Gan cao* 甘草	1. Glycyrrhiza uralensis Fisch. 2. Glycyrrhiza inflata Bat. 3. Glycyrrhiza glabra L	493 (27.21)
*Dang shen* 党参	1. Codonopsis pilosula (Franch.)Nannf. 2. Codonopsis pilosula Nannf.var.modesta (Nannf.) L.T.Shen 3. Codonopsis tangshenOliv.	489 (26.99)
*Qiang huo* 羌活	1. Notopterygium incisum Ting ex H. T. Chang 2. Notopterygium franchetii H. de Boiss.	467 (25.77)
*Shan zhu yu* 山茱萸	Cornus officinalis Sieb. et Zucc.	407 (22.46)
*Chai hu* 柴胡	1. Bupleurum chinenseDC. 2. Bupleurum scorzonerifolium Willd.	388 (21.41)
*Dang gui* 当归	Angelica sinensis (Oliv.) Diels	376 (20.75)
*Ge gen* 葛根	Pueraria lobata (Willd.) Ohwi	346 (19.09)
*Zhi qiao* 枳壳	Citrus aurantium L.	328 (18.10)
*Man jing zi* 蔓荆子	1. Vitex trifolia L. var. simplicifolia Cham. 2. Vitex trifolia L.	288 (15.89)
*Tian ma* 天麻	Gastrodia elata Bl.	287 (15.84)

No., number.

**FIGURE 2:**
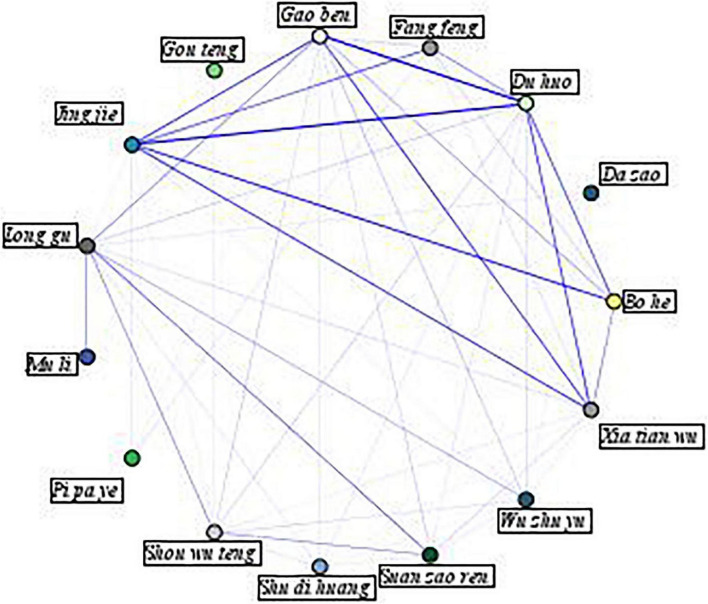
Network diagram of association rules between herbs.

To visualize the associations between individual herbs, a network diagram was generated and presented in Figure 2. In this figure, a thicker line indicates a stronger association between two herbs, indicating that these herbs are more commonly prescribed together. As the figure illustrates, the top 10 strongest links are *jing jie* and *du huo, gao ben*, and *du huo, bo he* and *jing jie, jing jie* and *xia tian wu, jing jie* and *gao ben, xia tian wu* and *du huo, xia tian wu* and *gao ben, bo he* and *du huo, fang feng* and *jing jie*, and *suan zao ren* and *long gu*.

##### Core herb pairs

To identify core herb pairs, 491 association rules were constructed when support was set as 5% and confidence as 50%, with only one antecedent. Among them, 15 herb pairs shared bidirectional associations with lifts over three (Table 5). Bidirectional associations are considered mandatory relationships (55, 56). The herb pair with the highest lift (that is, these herbs are more commonly prescribed together) is *bo he* and *jing jie*, followed by *jing jie* and *du huo, jing jie* and *fang feng, du huo* and *gao ben, du huo* and *bo he, du huo*, and *fang feng*.

**TABLE 5:** Core herb pairs in Chinese herbal decoctions for which patients reported improvements.

Consequent	Antecedent	Support %	Confidence %	Lift
*Bo he* 薄荷	*Jing jie* 荆芥	5.85	85.85	12.25
*Jing jie* 荆芥	*Bo he* 薄荷	7.01	71.65	12.25
*Jing jie* 荆芥	*Du huo* 独活	5.13	70.97	12.13
*Du huo* 独活	*Jing jie* 荆芥	5.85	62.26	12.13
*Fang feng* 防风	*Jing jie* 荆芥	5.85	93.40	11.43
*Jing jie* 荆芥	*Fang feng* 防风	8.17	66.89	11.43
*Gao ben* 藁本	*Du huo* 独活	5.13	59.14	11.28
*Du huo* 独活	*Gao ben* 藁本	5.24	57.89	11.28
*Bo he* 薄荷	*Du huo* 独活	5.13	70.97	10.13
*Du huo* 独活	*Bo he* 薄荷	7.01	51.97	10.13
*Fang feng* 防风	*Du huo* 独活	5.13	80.65	9.87
*Du huo* 独活	*Fang feng* 防风	8.17	50.68	9.87
*Jing jie* 荆芥	*Xia tian wu* 夏天无	5.57	56.44	9.65
*Xia tian wu* 夏天无	*Jing jie* 荆芥	5.85	53.77	9.65
*Pi pa ye* 枇杷叶	*Fang feng* 防风	8.17	84.46	9.11
*Fang feng* 防风	*Pi pa ye* 枇杷叶	9.27	74.40	9.11
*Fang feng* 防风	*Bo he* 薄荷	7.01	71.65	8.77
*Bo he* 薄荷	*Fang feng* 防风	8.17	61.49	8.77
*Sheng ma* 升麻	*Du zhong* 杜仲	7.28	63.64	7.21
*Du zhong* 杜仲	*Sheng ma* 升麻	8.83	52.50	7.21
*Xi xin* 细辛	*Fang feng* 防风	8.17	66.89	6.55
*Fang feng* 防风	*Xi xin* 细辛	10.21	53.51	6.55
*Ya jiao ai* 鸭脚艾	*Xiao hui xiang* 小茴香	29.58	95.52	3.17
*Xiao hui xiang* 小茴香	*Ya jiao ai* 鸭脚艾	30.13	93.77	3.17
*Ya jiao ai* 鸭脚艾	*Sha ren* 砂仁	29.03	92.21	3.06
*Sha ren* 砂仁	*Ya jiao ai* 鸭脚艾	30.13	88.83	3.06
*Xiao hui xiang* 小茴香	*Sha ren* 砂仁	29.03	90.30	3.05
*Sha ren* 砂仁	*Xiao hui xiang* 小茴香	29.58	88.62	3.05
*Hou po* 厚朴	*Zhi qiao* 枳壳	18.10	87.20	3.00
*Zhi qiao* 枳壳	*Hou po* 厚朴	29.03	54.37	3.00

The support level was set as 5%, confidence level as 50%.

Bidirectional associations with lift over 3.0 were presented.

##### Core herb combinations

During the iterative tests of the association rule for core herb combinations, it was found that the overall confidence in the association rules was high. Therefore, the confidence was preset at 95%, while the support value remained at 5%. The maximum number of antecedents was limited to eight. Based on the predetermined values (see “Materials and methods” section), a total of 837,008 association rules were constructed, 87 of which had a lift over 10 (Table 6). Only 17 herbs were involved in these 87 combinations since the combinations shared the same or similar herbs. The first combination covering the most overlapping herbs is similar to the formula *chuan xiong cha tiao san* (CXCTS), whose ingredients include *bo he, jing jie, fang feng, qiang huo, bai zhi*, and *chuan xiong*. The second combination includes *sheng ma, huang qi, man jing zi, ge gen, gan cao, bai zhu, fu ling, ze xie*, and *yuan zhi*, which is similar to the CHM formula *yi qi cong ming tang* (YQCMT).

**TABLE 6:** Core herb combinations in Chinese herbal decoctions for which patients reported improvements.

Consequent	Antecedent	Support %	Confidence %	Lift
*Jing jie* 荆芥	*Bo he* 薄荷 and *fang feng* 防风	5.02	98.90	16.91
*Jing jie* 荆芥	*Bo he* 薄荷 and *fang feng* 防风 and *qiang huo* 羌活	5.02	98.90	16.91
*Jing jie* 荆芥	*Bo he* 薄荷 and *fang feng* 防风 and *chuan xiong* 羌活	5.02	98.90	16.91
*Jing jie* 荆芥	*Bo he* 薄荷 and *qiang huo* 羌活 and *bai zhi* 白芷	5.02	98.90	16.91
*Jing jie* 荆芥	*Bo he* 薄荷 and *fang feng* 防风 and *qiang huo* 羌活 and *chuan xiong* 川芎	5.02	98.90	16.91
*Jing jie* 荆芥	*Bo he* 薄荷 and *qiang huo* 羌活 and *bai zhi* 白芷 and *chuan xiong* 川芎	5.02	98.90	16.91
*Jing jie* 荆芥	*Bo he* 薄荷 and *qiang huo* 羌活	5.13	96.77	16.54
*Jing jie* 荆芥	*Bo he* 薄荷 and *qiang huo* 羌活 and *chuan xiong* 川芎	5.13	96.77	16.54
*Bo he* 薄荷	*Jing jie* 荆芥 and *fang feng* 防风 and *qiang huo* 羌活	5.13	96.77	13.81
*Bo he* 薄荷	*Jing jie* 荆芥 and *fang feng* 防风 and *bai zhi* 白芷	5.13	96.77	13.81
*Bo he* 薄荷	*Jing jie* 荆芥 and *fang feng* 防风 and *qiang huo* 羌活 and *bai zhi* 白芷	5.13	96.77	13.81
*Bo he* 薄荷	*Jing jie* 荆芥 and *fang feng* 防风 and *qiang huo* 羌活 and *chuan xiong* 川芎	5.13	96.77	13.81
*Bo he* 薄荷	*Jing jie* 荆芥 and *fang feng* 防风 and *bai zhi* 白芷 and *chuan xiong* 川芎	5.13	96.77	13.81
*Bo he* 薄荷	*Jing jie* 荆芥 and *fang feng* 防风 and *qiang huo* 羌活 and *bai zhi* 白芷 and *chuan xiong* 川芎	5.13	96.77	13.81
*Bo he* 薄荷	*Jing jie* 荆芥 and *bai zhi* 白芷 and *chuan xiong* 川芎	5.19	95.74	13.66
*Bo he* 薄荷	*Jing jie* 荆芥 and *qiang huo* 羌活 and *bai zhi* 白芷 and *chuan xiong* 川芎	5.19	95.74	13.66
*Fang feng* 防风	*Pi pa ye* 枇杷叶 and *qiang huo* 羌活	5.52	99.00	12.12
*Fang feng* 防风	*Pi pa ye* 枇杷叶 and *qiang huo* 羌活 and *chuan xiong* 川芎	5.41	98.98	12.12
*Fang feng* 防风	*Jing jie* 荆芥 and *bai zhi* 白芷 and *chuan xiong* 川芎	5.19	98.94	12.11
*Fang feng* 防风	*Jing jie* 荆芥 and *qiang huo* 羌活 and *bai zhi* 白芷 and *chuan xiong* 川芎	5.19	98.94	12.11
*Fang feng* 防风	*Jing jie* 荆芥 and *Bo he* 薄荷	5.02	98.90	12.11
*Fang feng* 防风	*Bo he* 薄荷 and *qiang huo* 羌活 and *bai zhi* 白芷	5.02	98.90	12.11
*Fang feng* 防风	*Bo he* 薄荷 and *qiang huo* 羌活 and *bai zhi* 白芷 and *chuan xiong* 川芎	5.02	98.90	12.11
*Fang feng* 防风	*Jing jie* 荆芥 and *chuan xiong* 川芎	5.46	97.98	12.00
*Fang feng* 防风	*Jing jie* 荆芥 and *bai zhi* 白芷	5.24	97.89	11.99
*Fang feng* 防风	*Jing jie* 荆芥 and *qiang huo* 羌活 and *bai zhi* 白芷	5.24	97.89	11.99
*Fang feng* 防风	*Jing jie* 荆芥 and *qiang huo* 羌活 and *chuan xiong* 川芎	5.24	97.89	11.99
*Fang feng* 防风	*Bo he* 薄荷 and *qiang huo* 羌活	5.13	97.85	11.98
*Fang feng* 防风	*Bo he* 薄荷 and *qiang huo* 羌活and *chuan xiong* 川芎	5.13	97.85	11.98
*Fang feng* 防风	*Jing jie* 荆芥 and *qiang huo* 羌活	5.30	96.88	11.86
*Sheng ma* 升麻	*Huang qi* 黄芪 and *yuan zhi* 远志 and *ge gen* 葛根	5.24	98.95	11.21
*Sheng ma* 升麻	*Huang qi* 黄芪 and *man jing zi* 蔓荆子 and *bai zhu* 白术	5.24	98.95	11.21
*Sheng ma* 升麻	*Huang qi* 黄芪 and *yuan zhi* 远志 and *ge gen* 葛根 and *fu ling* 茯苓	5.24	98.95	11.21
*Sheng ma* 升麻	*Ze xie* 泽泻 and *Huang qi* 黄芪 and *yuan zhi* 远志	5.13	98.92	11.20
*Sheng ma* 升麻	*Ze xie* 泽泻 and *yuan zhi* 远志 and *man jing zi* 蔓荆子	5.13	98.92	11.20
*Sheng ma* 升麻	*Huang qi* 黄芪 and *yuan zhi* 远志 and *man jing zi* 蔓荆子	5.13	98.92	11.20
*Sheng ma* 升麻	*yuan zhi* 远志 and *man jing zi* 蔓荆子 and *fu ling* 茯苓	5.13	98.92	11.20
*Sheng ma* 升麻	*Ze xie* 泽泻 and *Huang qi* 黄芪 and *yuan zhi* 远志 and *man jing zi* 蔓荆子	5.13	98.92	11.20
*Sheng ma* 升麻	*Ze xie* 泽泻 and *Huang qi* 黄芪 and *yuan zhi* 远志 and *ge gen* 葛根	5.13	98.92	11.20
*Sheng ma* 升麻	*Ze xie* 泽泻 and *Huang qi* 黄芪 and *yuan zhi* 远志 and *fu ling* 茯苓	5.13	98.92	11.20
*Sheng ma* 升麻	*Ze xie* 泽泻 and *yuan zhi* 远志 and *man jing zi* 蔓荆子 and *ge gen* 葛根	5.13	98.92	11.20
*Sheng ma* 升麻	*Ze xie* 泽泻 and *yuan zhi* 远志 and *man jing zi* 蔓荆子 and *fu ling* 茯苓	5.13	98.92	11.20
*Sheng ma* 升麻	*Huang qi* 黄芪 and *yuan zhi* 远志 and *man jing zi* 蔓荆子 and *ge gen* 葛根	5.13	98.92	11.20
*Sheng ma* 升麻	*Huang qi* 黄芪 and *yuan zhi* 远志 and *man jing zi* 蔓荆子 and *fu ling* 茯苓	5.13	98.92	11.20
*Sheng ma* 升麻	*yuan zhi* 远志 and *man jing zi* 蔓荆子 and *ge gen* 葛根 and *fu ling* 茯苓	5.13	98.92	11.20
*Sheng ma* 升麻	*Ze xie* 泽泻 and *Huang qi* 黄芪 and *yuan zhi* 远志 and *man jing zi* 蔓荆子 and *ge gen* 葛根	5.13	98.92	11.20
*Sheng ma* 升麻	*Ze xie* 泽泻 and *Huang qi* 黄芪 and *yuan zhi* 远志 and *man jing zi* 蔓荆子 and *fu ling* 茯苓	5.13	98.92	11.20
*Sheng ma* 升麻	*Ze xie* 泽泻 and *Huang qi* 黄芪 and *yuan zhi* 远志 and *ge gen* 葛根 and *fu ling* 茯苓	5.13	98.92	11.20
*Sheng ma* 升麻	*Ze xie* 泽泻 and *yuan zhi* 远志 and *man jing zi* 蔓荆子 and *ge gen* 葛根 and *fu ling* 茯苓	5.13	98.92	11.20
*Sheng ma* 升麻	*Huang qi* 黄芪 and *yuan zhi* 远志 and *man jing zi* 蔓荆子 and *ge gen* 葛根 and *fu ling* 茯苓	5.13	98.92	11.20
*Sheng ma* 升麻	*Ze xie* 泽泻 and *Huang qi* 黄芪 and *yuan zhi* 远志 and *man jing zi* 蔓荆子 and *ge gen* 葛根 and *fu ling* 茯苓	5.13	98.92	11.20
*Sheng ma* 升麻	*Huang qi* 黄芪 and *man jing zi* 蔓荆子 and *ge gen* 葛根 and *bai zhu* 白术	5.08	98.91	11.20
*Sheng ma* 升麻	*Huang qi* 黄芪 and *man jing zi* 蔓荆子 and *bai zhu* 白术 and *fu ling* 茯苓	5.08	98.91	11.20
*Sheng ma* 升麻	*Huang qi* 黄芪 and *man jing zi* 蔓荆子 and *ge gen* 葛根 and *bai zhu* 白术 and *fu ling* 茯苓	5.08	98.91	11.20
*Sheng ma* 升麻	*yuan zhi* 远志 and *man jing zi* 蔓荆子	5.19	97.87	11.08
*Sheng ma* 升麻	*yuan zhi* 远志 and *man jing zi* 蔓荆子and *ge gen* 葛根	5.19	97.87	11.08
*Sheng ma* 升麻	*man jing zi* 蔓荆子 and *ge gen* 葛根 and *bai zhu* 白术 and *fu ling* 茯苓	5.13	97.85	11.08
*Sheng ma* 升麻	*Huang qi* 黄芪 and *man jing zi* 蔓荆子 and *ge gen* 葛根 and *fu ling* 茯苓	5.68	97.09	11.00
*Sheng ma* 升麻	*Ze xie* 泽泻 and *Huang qi* 黄芪 and *ge gen* 葛根	5.52	97.00	10.99
*Sheng ma* 升麻	*Ze xie* 泽泻 and *Huang qi* 黄芪 and *man jing zi* 蔓荆子	5.46	96.97	10.98
*Sheng ma* 升麻	*Ze xie* 泽泻 and *man jing zi* 蔓荆子 and *ge gen* 葛根	5.46	96.97	10.98
*Sheng ma* 升麻	*Ze xie* 泽泻 and *Huang qi* 黄芪 and *man jing zi* 蔓荆子 and *ge gen* 葛根	5.46	96.97	10.98
*Sheng ma* 升麻	*Huang qi* 黄芪 and *ge gen* 葛根 and *dang gui* 当归 and *fu ling* 茯苓	5.46	96.97	10.98
*Sheng ma* 升麻	*Ze xie* 泽泻 and *Huang qi* 黄芪 and *ge gen* 葛根 and *fu ling* 茯苓	5.41	96.94	10.98
*Sheng ma* 升麻	*Ze xie* 泽泻 and *Huang qi* 黄芪 and *man jing zi* 蔓荆子 and *fu ling* 茯苓	5.35	96.91	10.97
*Sheng ma* 升麻	*Ze xie* 泽泻 and *man jing zi* 蔓荆子 and *ge gen* 葛根 and *fu ling* 茯苓	5.35	96.91	10.97
*Sheng ma* 升麻	*Ze xie* 泽泻 and *Huang qi* 黄芪 and *man jing zi* 蔓荆子 and *ge gen* 葛根 and *fu ling* 茯苓	5.35	96.91	10.97
*Sheng ma* 升麻	*Huang qi* 黄芪 and *man jing zi* 蔓荆子 and *ge gen* 葛根 and *dang gui* 当归 and *fu ling* 茯苓	5.30	96.88	10.97
*Sheng ma* 升麻	*Huang qi* 黄芪 and *yuan zhi* 远志 and *dang gui* 当归 and *jiu jie chang pu* 九节菖蒲	5.13	96.77	10.96
*Sheng ma* 升麻	*Huang qi* 黄芪 and *man jing zi* 蔓荆子 and *ge gen* 葛根 and *gan cao* 甘草	5.13	96.77	10.96
*Sheng ma* 升麻	*Huang qi* 黄芪 and *yuan zhi* 远志 and *dang gui* 当归 and *jiu jie chang pu* 九节菖蒲 and *fu ling* 茯苓	5.13	96.77	10.96
*Sheng ma* 升麻	*Huang qi* 黄芪 and *man jing zi* 蔓荆子 and *ge gen* 葛根 and *gan cao* 甘草 and *fu ling* 茯苓	5.02	96.70	10.95
*Sheng ma* 升麻	*Huang qi* 黄芪 and *ge gen* 葛根 and *dang gui* 当归	5.52	96.00	10.87
*Sheng ma* 升麻	*Huang qi* 黄芪 and *man jing zi* 蔓荆子 and *ge gen* 葛根 and *dang gui* 当归	5.35	95.88	10.86
*Sheng ma* 升麻	*man jing zi* 蔓荆子 and *ge gen* 葛根 and *dang gui* 当归 and *fu ling* 茯苓	5.35	95.88	10.86
*Sheng ma* 升麻	*Ze xie* 泽泻 and *yuan zhi* 远志 and *ge gen* 葛根	5.30	95.83	10.85
*Sheng ma* 升麻	*Ze xie* 泽泻 and *yuan zhi* 远志 and *ge gen* 葛根 and *fu ling* 茯苓	5.30	95.83	10.85
*Sheng ma* 升麻	*Ze xie* 泽泻 and *ge gen* 葛根 and *dang gui* 当归	5.08	95.65	10.83
*Sheng ma* 升麻	*Ze xie* 泽泻 and *ge gen* 葛根 and *dang gui* 当归 and *fu ling* 茯苓	5.08	95.65	10.83
*Sheng ma* 升麻	*Huang qi* 黄芪 and *man jing zi* 蔓荆子 and *gan cao* 甘草 and *fu ling* 茯苓	5.08	95.65	10.83
*Sheng ma* 升麻	*Huang qi* 黄芪 and *ge gen* 葛根 and *gan cao* 甘草 and *fu ling* 茯苓	5.08	95.65	10.83
*Sheng ma* 升麻	*Huang qi* 黄芪 and *yuan zhi* 远志 and *bai zhu* 白术	5.02	95.60	10.83
*Sheng ma* 升麻	*Huang qi* 黄芪 and *yuan zhi* 远志 and *bai zhu* 白术 and *fu ling* 茯苓	5.02	95.60	10.83
*Sheng ma* 升麻	*Huang qi* 黄芪 and *yuan zhi* 远志 and *dang gui* 当归	5.57	95.05	10.76
*Sheng ma* 升麻	*Huang qi* 黄芪 and *yuan zhi* 远志 and *dang gui* 当归 and *fu ling* 茯苓	5.57	95.05	10.76
*Sheng ma* 升麻	*Huang qi* 黄芪 and *dang gui* 当归 and *jiu jie chang pu* 九节菖蒲	5.52	95.00	10.76
*Sheng ma* 升麻	*Huang qi* 黄芪 and *dang gui* 当归 and *jiu jie chang pu* 九节菖蒲 and *fu ling* 茯苓	5.52	95.00	10.76

Support level was set at 5%, and confidence level at 95%.

Associations with lift ≥ 10 were presented.

##### Associations between comorbidities/triggers and herbs

Triggers and comorbidities were set as antecedents, and frequently used herbs were put in the consequent sets. The support level was preset as 5% and confidence as 50%. Constructed associations with lift ≥ 2.0 are presented in Table 7. Menstruation was the only trigger successfully included in the associations and was usually associated with herbs that activate the circulation of Blood and *qi*, such as *xiao hui xiang, wu yao, sha ren*, and *xiang fu*.

**TABLE 7:** Association rules between comorbidities/triggers and herbs.

Consequent	Antecedent	Support %	Confidence %	Lift	Functions of the herb
*Xiao hui xiang* 小茴香	Menstruation	14.96	67.90	2.30	Expelling the Coldness and warming the meridian, alleviating pain, activating the circulation of *qi*
*Wu yao* 乌药	Menstruation	14.96	76.75	2.23	Activating the circulation of *qi* and alleviating pain, expelling the Coldness, and warming the Kidney
*Sha ren* 砂仁	Menstruation	14.96	64.58	2.22	Eliminating dampness and activating the circulation of *qi*
*Ya jiao ai* 鸭脚艾	Menstruation	14.96	66.79	2.22	Activating the circulation of Blood and removing the Stasis of Blood, smoothing the meridians, and alleviating the pain
*Xiang fu* 香附	Menstruation	14.96	69.00	2.04	Smoothing the Liver meridian, activating the circulation of *qi* and Blood, and alleviating the pain
*Chen pi* 陈皮	Menstruation	14.96	82.29	2.03	Tonifying the Spleen, activating the circulation of *qi* and eliminating Dampness and Phlegm

The support level was set as 5%, confidence level as 50%.

Associations with lift ≥ 2.0 were presented.

#### Patented Chinese herbal medicine products

A total of 51 PCHMPs were identified from the 1,158 PERIs that used PCHMPs. The top 14 most frequently used PCHMPs are presented in Table 8. The leading PCHMP was *tong tian* oral solution, with a frequency of 762 (65.80%). It consists of *chuan xiong, chi shao, tian ma, qiang huo, bai zhi, xi xin, ju hua, bo he, fang feng, cha*, and *gan cao*. The other PCHMPs that contributed to more than 15% of PERIs were *jian wei yu yang* tablet (*n* = 208, 17.96%), *tian shu* tablet (*n* = 206, 17.79%), and *er shi wu wei shan hu* capsule (*n* = 196, 16.93%).

**TABLE 8:** Frequently used patented Chinese herbal medicine products for which patients reported improvements.

Names in *pinyin* and Chinese characters	No. (%) (Total valid No. = 1,158)	Ingredients
*Tong tian* oral solution 通天口服液	762 (65.80)	*chuan xiong* 川芎, *chi shao* 赤芍, *tian ma* 天麻, *qiang huo* 羌活, *bai zhi* 白芷, *xi xin* 细辛, *ju hua* 菊花, *bo he* 薄荷, *fang feng* 防风, *cha* 茶, *gan cao* 甘草
*Jian wei yu yang* tablet 健胃愈疡片	208 (17.96)	*chai hu* 柴胡, *dang shen* 党参, *bai shao* 白芍, *yan hu suo* 延胡素, *bai ji* 白芨, *zhen zhu ceng fen* 珍珠层粉, *qing dai* 青黛, *gan cao* 甘草
*Tian shu* tablet 天舒片	206 (17.79)	*chuan xiong* 川芎, *tian ma* 天麻
*Er shi wu wei shan hu* capsule 二十五味珊瑚丸	196 (16.93)	*shan hu* 珊瑚, *pearl* 珍珠, *qing jin shi* 青金石, *zhen zhu mu* 珍珠母, *he zi* 诃子, *mu xiang* 木香, *hong hua* 红花, *ding xiang* 丁香, *chen xiang* 沉香, *zhu sha* 朱砂, *long gu* 龙骨, *lu gan shi* 炉甘石, *nao shi* 脑石, *ci shi*磁石, *yu liang tu*禹粮土, *zhi ma* 芝麻, *hu lu* 葫芦, *zi yuan* 紫菀花, *zhang ya cai* 獐牙菜, *chang pu* 菖蒲, *bang na* 榜那, *da jian ju* 打箭菊, *gan cao* 甘草, *hong hua* 红花, *she xiang* 麝香
*Zao ren an shen* capsule 枣仁安神胶囊	126 (10.88)	*suan zao ren* 酸枣仁, *dan shen* 丹参, *wu wei zi* 五味子
*Wei su* granule 胃苏颗粒	120 (10.36)	*zi su* 紫苏, *xiang fu* 香附, *chen pi* 陈皮, *xiang yuan* 香橼, *fo shou* 佛手, *zhi qiao* 枳壳, *bing lang* 槟榔, *ji nei jin* 鸡内金
*Yang xue qing nao* granule 养血清脑颗粒	94 (8.12)	*dang gui* 当归, *chuan xiong* 川芎, *bai shao* 白芍, *shu di huang* 熟地黄, *gou teng* 钩藤, *ji xue teng* 鸡血藤, *xia ku cao* 夏枯草, *jue ming zi* 决明子, *zhen zhu mu* 珍珠母, *yan hu suo* 延胡索, *xi xin* 细辛
*Deng zhan sheng mai* capsule 灯盏生脉胶囊	34 (2.94)	*deng zhan xi xin* 灯盏细辛, *ren shen* 人参, *wu wei zi* 五味子, *mai dong* 麦冬
*San qi tong shu* capsule 三七通舒胶囊	32 (2.76)	*san qi* 三七
*Song ling xue mai kang* capsule 松龄血脉康胶囊	26 (2.25)	*song ye* 松叶, *ge gen* 葛根, *pearl powder* 珍珠粉
*Wu ling* capsule 乌灵胶囊	26 (2.25)	*wu ling jun powder* 乌灵菌粉
*Zha chong shi san wei* pill 扎冲十三味丸	26 (2.25)	*he zi* 诃子, *cao wu* 草乌, *shi chang pu* 石菖蒲, *mu xiang* 木香, *she xiang* 麝香, *shan hu* 珊瑚, *pearl* 珍珠, *ding xiang* 丁香, *rou dou kou* 肉豆蔻, *chen xiang* 沉香, *yu liang tu* 禹余粮土, *ci shi* 磁石, *gan cao* 甘草
*Tian dan tong luo* capsule 天丹通络胶囊	25 (2.16)	*chuan xiong* 川芎, *xi qian cao* 豨签草, *dan shen* 丹参, *shui zhi* 水蛭, *tian ma* 天麻, *huai hua* 槐花, *shi chang pu* 石菖蒲, *niu huang* 牛黄, *huang qi* 黄芪, *niu xi* 牛膝
*Xue fu zhu yu* oral solution/tablet 血府逐瘀口服液/片	23 (1.99)	*tao ren* 桃仁, *hong hua* 红花, *dang gui* 当归, *chuan xiong* 川芎, *di huang* 地黄, *chi shao* 赤芍, *niu xi* 牛膝, *chai hu* 柴胡, *zhi qiao* 枳壳, *jie geng* 桔梗, *gan cao* 甘草

No., number.

## Mechanism of herbs and formula

The most frequently used herb, *fu ling*, tonifies Spleen and eliminates dampness (50). Patients in this study are primarily located in southern China, and as such, dampness would be the key Chinese medicine pathogenic factor in their cases (57, 58). It could be explained that *fu ling* was often used due to these patients’ general constitutions. Currently, there is no direct pre-clinical evidence to support *fu ling*’s mechanism for curing migraine or headaches. However, research illustrated that Poria cocos polysaccharide, an active compound of *fu ling*, could exert neuroprotective effects by alleviating oxidative stress, apoptosis, inflammation, and inhibiting the MAPK/NF-κB pathway in Alzheimer’s disease rats (59). Similar pathways were identified in migraine pathology (60–62).

The second frequently used herb *chuan xiong* activates *qi* and Blood, expels Wind, and alleviates pain (50). It has been widely used for migraine and headaches in historical and current clinical practice (23, 63–65). An RCT-based systematic review indicated that formulae containing *chuan xiong* were effective in migraine prophylaxis (24). The key active compound of *chuan xiong*, Senkyunolide I, exerts anti-migraine effects by adjusting monoamine neurotransmitters levels and turnover rates and decreases nitric oxide levels in the blood and brain (66). Also, the volatile oil from *chuan xiong* presents an analgesic effect by inhibiting the *c*-fos gene expression and plasma CGRP in nitroglycerin-induced headaches in rats (67). In addition, a *chuan xiong* extract, ligustrazine, showed potent activity against nitroglycerin-induced migraine in rats by inhibiting the *c*-fos/ERK signaling pathway (68).

The formula CXCTS has also been widely used in historical and current clinical practice (69, 70), and has been proven effective for migraine management (71). CXCTS exerts anti-migraine effects by reducing the CGRP level (72) and inhibiting the PI3K-AKT and HIF-1 signaling pathways (73).

The formula YQCMT was proved to be more effective than flunarizine in treating vestibular migraine with a Chinese medicine syndrome of *qi* deficiency (74). Mechanisms on the core herb pairs for migraine are yet to be discovered and require further bench research.

## Discussion

### Summary of the results

Migraine is a prevalent, disabling disease that causes significant burdens on patients and the health system. As current conventional migraine management, including pharmacotherapies and lifestyle changes, is not always effective, it is important to explore how complementary and alternative treatments for migraines can be used in real-world clinical practice. EMRs are invaluable sources of real-world data that can be used to generate clinical practice evidence (75). Hospital-based EMRs have the natural advantages of being reliable sources, large sample sizes, structured frameworks, and diverse levels (76). This real-world clinical data provides first-hand, convincing information about Chinese medicine clinicians’ experience (77), which can vitally contribute to evidence-based practice (78).

This retrospective study summarized migraine patients’ characteristics, comorbidities, and triggers based on EMRs from a tertiary Chinese medicine hospital with approximately seven million outpatient visits annually. The gender distribution of all EMRs (female to male ratio 3.4:1), among patients receiving CHM (1,395:395) and acupuncture-related treatments (264:84), are all consistent with previous epidemiological studies (79, 80). The age range of patients is also consistent with the Global Burden of Disease Study (9). The comorbidities (e.g., sleep disorders, anxiety, and depression) and triggers (e.g., fatigue, stress, diet, poor sleep, and menstruation) identified in this study have been commonly reported in previous research (2–5, 81–85). This study found that fatigue is the most commonly reported trigger of migraine attacks. However, fatigue might be a prodromal symptom (86) rather than a trigger, and migraine patients might not be able to differentiate between prodromal symptoms and triggers. In addition, it should be acknowledged that the information on triggers collected from EMRs was not as accurate as those recorded in patients’ migraine diaries used in clinical trials.

This study also summarized the treatment patterns in the hospital. It was found that CHM was more frequently prescribed to migraine patients than acupuncture and conventional pharmacotherapies, without significant differences between gender or age groups (adult group vs. non-adult group). In addition, we found that the rate of acute medication-taking history among migraine patients in the Chinese medicine hospital was relatively low (21.01%), especially when compared to the rate of acute medication usage in the U.S. [from 26% (12) to 95.1% (80)], Australia (80%) (87), and Japan (73%) (88). The proportion of patients being newly prescribed acute medication was also much lower (1.58%). While the proportion of patients being prescribed prophylactic medication (mainly CCBs, 21.16%) in the hospital is lower than that in the U.S. (30–60%) (89, 90). Such a low rate of conventional pharmacotherapies recorded in these EMRs could be due to (1) people purchasing over-the-counter acute medications and therefore these were not recorded in the EMRs; and/or (2) patients being given pharmacotherapies by other doctors before seeking additional Chinese medicine treatments. It is worth investigating whether taking CHM can reduce the usage of acute medication in rigorously designed RCTs in future because less acute medication usage can avoid the potential risk of medication overuse.

Our study briefly evaluated the overall improvement rate (50.42%). This was comparable to flunarizine, which has a commonly reported improvement rate ranging from 46.15 to 66.7% (91, 92). However, it should be noted that this was based on patient-reported improvements in all aspects, including the reduction of migraine or other symptoms in general. This was not accurately measured by the standard quantitative outcome measures used in clinical trials. It is also worth noting that improvements in comorbidities, such as insomnia and anxiety, were reported, although anti-depressant and hypnotic drugs were rarely used. Such findings could indicate the potential effects of Chinese medicine in managing various comorbidities and requires further exploration in future studies.

The treatment duration for migraine in the Chinese hospital (mean = 10 days) is shorter than the recommended duration of “at least 4 weeks” for conventional pharmacotherapies (10, 11). The duration for patients to report general improvements is also relatively short (median duration = 14 days), though not rigorously measured. This raised our curiosity about the optimal treatment duration and long-term effects of Chinese medicine. Whether adding Chinese medicine therapies to migraine management could shorten the required treatment duration is yet to be discovered.

Most patients were treated with a single type of treatment, mainly CHM (53.58%) or acupuncture (7.22%). Although both monotherapy and combination therapies of Chinese medicine with conventional pharmacotherapies have been proven effective for migraine in clinical trials (23–25), we suggest a pragmatic trial to compare the therapeutic effects and economic cost of Chinese medicine alone to that of Chinese medicine combined with conventional pharmacotherapies.

Furthermore, the study provided real-world clinicians’ experience of prescribing CHM for migraine. The frequency analysis results revealed that the most frequently used herbs are *fu ling* and *chuan xiong*, the most frequently used formula is CXCTS, and PCHMP is *tong tian* oral solution. These herbs and formulae have been proven to carry potential anti-migraine effects in previous bench or clinical research.

Identifying core CHM treatments is essential for selecting candidates for basic research, clinical trials, and daily practice (93, 94). Herb pair is the smallest compatible unit in CHM formulae, referring to two individual herbs repeatedly coexisting to enhance therapeutic effects or reduce toxicity (95). The top herb pair with a bidirectional association is *bo he* and *jing jie*. The core formulae found by herb combination construction include CXCTS and YQCMT, while CXCTS is also the basic formula for *tong tian* oral solution. These formulae are recommended for migraine treatment in the Guidelines for Diagnosis and Treatment of Common Internal Diseases in Chinese Medicine (96).

The constructed association rules between comorbidities/triggers and herbs indicate that females with menstruation-triggered migraine are more likely to be prescribed herbs that warm the meridian and activate the circulation of Blood and *qi*. This is consistent with recently published data-mining results (97). These results provide insight into the management of menstrual migraine and menstrually related migraine. We did not conduct association rule construction between other health conditions (e.g., hypertension and diabetes) and herbs, because (1) these conditions are not closely associated with migraine; and (2) they were not detailed in the EMRs for patients who visited the hospital to seek migraine treatment.

### Limitations

The findings of this study should be interpreted with several limitations in mind. First, the patients’ characteristics, triggers, and comorbidities were collected from the EMRs, then there is a lack of consistency in depth and detail across EMRs. Second, the treatment response was defined and extracted based on the text recorded in the EMRs. It was not feasible for us to rigorously evaluate the effectiveness, because clinicians did not record treatment effects assessed by standard outcome measures as is done in clinical trials. Third, this study was conducted in one Chinese medicine hospital, so the results may be restricted in generality and more valuable for Chinese medicine clinical practice in southern China. Multi-center, prospective registry studies based on different geographic locations will provide more accurate and applicable information about Chinese medicine treatments for migraine.

## Conclusion

This study presented the clinical features of 2,023 migraine patients and their treatment patterns, based on their EMRs in a Chinese medicine hospital. CHM can be used as an alternative to conventional pharmacotherapies, given that CHM was taking predominant treatment for migraine management while acute medication and prophylactic medicine were only prescribed to a small proportion of the migraine patients in the hospital. CHM formulae, such as *chuan xiong cha tiao san* and *yi qi cong ming tang*, patented CHM product *tong tian* oral solution, and some herb ingredients are potentially effective for migraine and are worth further evaluation. The optimal treatment duration, long-term effects, and treatment-effect curve of Chinese medicine for migraine need further exploration.

## Data availability statement

The original contributions presented in this study are included in the article/supplementary material, further inquiries can be directed to the corresponding author/s.

## Author contributions

SL and CZ planned and drafted the article. CZ, AZ, JS, XG, and CX provided the informative and critical comments on the manuscript revision. SL and HW extracted and screened the data. SL conducted the data analyses. SL, XG, and AZ undertook the final proofing of the manuscript and are responsible for its accuracy. All authors critically revised the manuscript and approved the final version.

## Abbreviations

CCB: calcium channel blocker
CGRP: calcitonin gene-related peptide
CHM: Chinese herbal medicine
CXCTS: formula of *chuan xiong cha tiao san*
PE: patient encounter
EMR: electronic medical record
GPHCM: Guangdong Provincial Hospital of Chinese Medicine
PERI: patient encounters reporting improvement
PCHMP: Patented Chinese herbal medicine products
YLD: years of life with disability
YQCMT: formula of *yi qi cong ming tang.*
